# Attempted Ovariotomy

**Published:** 1871-02

**Authors:** 


### Attempted Ovariotomy.

Dr. Jouon, professor at the medical school at Nantes, in France, being called upon to perform ovariotomy upon a married woman of twenty-nine, observed, after his incision from below the umbilicus to the pubes, and the puncturing of the cyst, that the adhesions were so tight that the sac could not be isolated. The idea of actual ovariotomy was then given up, and the case treated as one of artificial anus. The margins of the wound were secured against the cyst, and a tent passed into the latter. The patient had a fearful reaction; but, by dint of attentive care, and the syringing of the cyst, first with alcohol and water, and afterward with tincture of iodine, with appropriate narcotic and tonic remedies, the woman was quite restored in a little less than four months.—*Lancet.*

